# Feasibility Study of Detecting and Segmenting Small Brain Tumors in a Small MRI Dataset with Self-Supervised Learning

**DOI:** 10.3390/diagnostics15030249

**Published:** 2025-01-22

**Authors:** Wei-Jun Zhang, Wei-Teing Chen, Chien-Hung Liu, Shiuan-Wen Chen, Yu-Hua Lai, Shingchern D. You

**Affiliations:** 1 Department of Computer Science and Information Engineering, National Taipei University of Technology, Taipei 106, Taiwan; justinzhang515@gmail.com (W.-J.Z.); cliu@ntut.edu.tw (C.-H.L.); 2 Division of Thoracic Medicine, Department of Medicine, Cheng Hsin General Hospital, Taipei 112, Taiwan; 3 Department of Internal Medicine, Tri-Service General Hospital, National Defense Medical Center, Taipei 114, Taiwan; 4 Department of Electrical and Computer Engineering (ECE), University of Toronto, Toronto, ON M5S 1A1, Canada; shiuanwen.chen@utoronto.ca; 5 Division of Neurology, Department of Medicine, Cheng Hsin General Hospital, Taipei 112, Taiwan; ch9419@chgh.org.tw

**Keywords:** brain tumor, deep learning model, brain MRI

## Abstract

**Objectives:** This paper studies the segmentation and detection of small metastatic brain tumors. This study aims to evaluate the feasibility of training a deep neural network for the segmentation and detection of metastatic brain tumors in MRI using a very small dataset of 33 cases, by leveraging large public datasets of primary tumors; **Methods:** This study explores various methods, including supervised learning, two transfer learning approaches, and self-supervised learning, utilizing U-net and Swin UNETR models; **Results:** The self-supervised learning approach utilizing the Swin UNETR model yielded the best performance. The Dice score for small brain tumors was approximately 0.19. Sensitivity reached 100%, while specificity was 54.5%. When excluding subjects with hyperintensities, the specificity improved to 80.0%; **Conclusions:** It is feasible to train a model using self-supervised learning and a small dataset for the segmentation and detection of small brain tumors.

## 1. Introduction

A brain tumor is an abnormal mass of cells in the brain that can develop at any stage of life. These cells divide and grow uncontrollably, occupying the limited and enclosed space of the brain or invading normal brain tissue, leading to various symptoms. This type of disease is relatively common, with approximately 250,000 new cases globally each year. In Australia, the government is investing AUD 126.4 million into brain cancer research. This investment is prompted by the incidence of around 2000 Australians being diagnosed with brain cancer every year and the low 5-year survival rate of approximately 23% [1].

Brain tumors are named based on their location and the classification of the tumor cells, which can be primary or metastatic, benign or malignant, and vary in size. Brain metastases and primary brain tumors with multiple foci glioblastoma (GBM) are two different pathophysiological entities requiring distinct therapeutic approaches. Primary brain tumors originate within the brain parenchyma itself, including glioma, meningioma, and astrocytoma. On the other hand, metastatic brain tumors originate from metastatic cells spreading from other parts of primary malignancy, such as lung, breast, and colorectal cancers [2,3].

Patients with primary or metastatic brain tumors exhibit different symptoms depending on the size, location, and distribution of the lesion within the brain. As the tumor grows within the limited space of the skull, it increases intracranial pressure and endangers the patient’s life. This may cause brain edema due to plasma-like fluid leakage through impaired capillary endothelial tight junctions in tumors [4]. The mass effect with midline shift, tumor dimensions, and mass-edema index may contribute to the differential diagnosis of metastatic versus primary brain metastasis [5].

The size of brain tumors can range from very small to very large with different progression. The prognosis of a patient depends on several factors, including the type of brain tumor and its growth rate [6]. Since growth speed is a major concern in treating brain tumors, it is best to detect them when they are still small. However, there are currently not many papers discussing how to detect small brain tumors.

To diagnose brain tumors, neurologists and neurosurgeons inquire about the patient’s and their family’s medical history and conduct a comprehensive neurological examination. This includes assessments of consciousness, muscle strength, coordination, reflexes, and pain response to identify the cause of symptoms. Progressive visual field defect and visual acuity loss generally occur due to an intracranial tumor [7].

Based on the results of physical and neurological examinations, further tests may be selected, such as a computer tomography (CT) scan, magnetic resonance imaging (MRI), electroencephalogram (EEG), cerebral angiography, and positron emission tomography (PET) [8]. In this paper, we focus on the use of MRI to detect and segment small tumors.

To reduce the cost of examinations by experts, automated detection and/or segmentation is desirable. In the field of automated brain tumor segmentation, many methods have been proposed, such as region-based approaches [9,10,11,12], edge-based approaches [11,13], threshold-based approaches [11,12], and deep learning-based approaches [12,14,15,16,17,18,19]. Additionally, several survey papers are available on these methods [11,20,21,22]. It can be observed that deep learning-based approaches are preferable for brain tumor segmentation, as evidenced by the vast number of recent publications [21,22]. Among the deep learning-based approaches, U-net [16] has been one of the preferred models due to its good segmentation performance. Recent studies by other scholars [17] indicate that adding the Transformer [23] module to the U-net architecture can effectively improve segmentation results.

Despite the great success of deep learning methods, these methods require a large number of labeled samples and the high cost of labeling, which typically requires professional doctors to invest significant time and effort. The high cost of tumor samples further limits the performance of supervised learning methods.

To mitigate this problem, several efforts have been made to create public brain MRI datasets for researchers, such as those in [24,25,26]. Among the datasets, the Brain Tumor Segmentation (BraTS) challenge 2021 dataset [26,27,28,29] is “utilized primarily by maximum researchers” [22]. With data from over 1200 cases with annotations, it is a highly valuable resource for segmenting brain tumors in MRI images. More details about the BraTS 2021 dataset will be provided in Section 3.1.1.

Although the BraTS 2021 dataset is available to the general public, it contains only cases of primary brain tumors with annotations. In contrast, to the best of our knowledge, there is no public 3D MRI dataset that includes cases of metastatic brain tumors. While both primary and metastatic brain tumors develop in brain tissues, they can be distinguished using MRI scans [30]. A previous study investigated the imaging differences between GBM and multiple brain metastases, aiming to develop a diagnostic algorithm for differentiation on initial MRI, utilizing apparent diffusion coefficient (ADC) values, surrounding T2-hyperintensity, and edema distribution.

**Figure 1:**
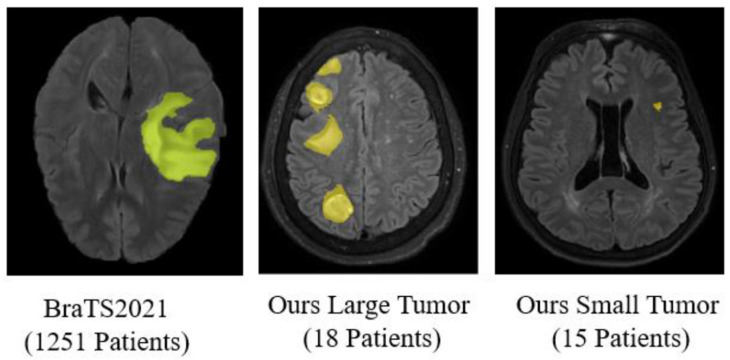
MRI Images of large and small tumors.

To assess the segmentation performance in real-world scenarios, particularly for metastatic brain tumors, we randomly collected MRI cases from Cheng Hsin General Hospital (CHGH) in Taipei, Taiwan. The selection was based solely on medical records without prior viewing of the MRI images, resulting in a dataset that includes both large and small tumors. The definitions of large and small tumors will be provided in Section 3.1.2. The visual distinction between large and small brain tumors is evident, as illustrated in Figure 1. Given the time-consuming and costly process of annotating tumor regions, our goal is to develop a feasible approach for segmenting and detecting small brain tumors with very limited available data, supplemented by a public dataset containing primary tumors. This approach presents challenges, as the imaging features of primary and metastatic tumors may differ.

In this paper, the training methods explored include supervised learning, two transfer learning approaches, and self-supervised learning. Unlike existing research, which typically focuses on either segmentation or detection performance, our objective is to identify the most effective segmentation method and subsequently evaluate its detection performance.

## 2. Related Works

Ronneberger et al. [16] proposed a neural network architecture called U-net, which is based on an autoencoder architecture. This architecture features skip connections that link the feature map of each encoder layer to the corresponding decoder layer’s feature map. These connections help the decoder retain more contextual information during upsampling, thereby improving the accuracy of segmentation results. It has also been demonstrated that the U-net architecture outperforms the previous best methods in the International Symposium on Biomedical Imaging (ISBI) challenge.

Hatamizadeh et al. [17] proposed a variant of U-net called Swin UNETR, which primarily uses the Swin Transformer [31] as the encoder. They added a residual network to the skip connections that link the encoder and decoder. The Swin UNETR architecture also achieved excellent results in the BraTS2021 segmentation challenge.

Chen et al. [32] proposed a framework for image contrastive learning called SimCLR, which simplifies previously proposed self-supervised learning algorithms. It demonstrates that the combination of data augmentation in contrastive learning plays a crucial role in prediction tasks. The SimCLR method has been proven to outperform previous self-supervised and semi-supervised learning methods, achieving good accuracy even with only 1% of the labeled training samples.

Tang et al. [33] proposed a self-supervised learning framework for 3D medical imaging, using the Swin Transformer encoder for contrastive learning, followed by fine-tuning segmentation tasks with Swin UNETR. The authors used their method to perform self-supervised pre-training on 5050 open-source CT images of different body organs, followed by fine-tuning on the Beyond the Cranial Vault (BTCV) and Medical Segmentation Decathlon (MSD) datasets. The results show that their model ranks first on the test leaderboards of the BTCV and MSD datasets.

Abdusalomov et al. used Yolov7 to detect gliomas, meningiomas, and pituitary brain tumors [19]. With a fairly large dataset (3400+ samples per plane) with data augmentation, they achieved very high detection sensitivity and specificity. However, this paper does not provide sufficient information about the size of the tumors in the study.

Mansur et al. [12] investigated brain tumor segmentation using three different methods: threshold-based, region-based, and CNN-based approaches. Their results indicated that the threshold-based approach outperformed the other two methods. However, they utilized the Kaggle dataset [25], which is a 2D dataset (i.e., one image per case), rather than a 3D dataset. Consequently, their findings cannot be directly applied to our study, which involves the use of 3D images.

Kaifi provided a comprehensive review of AI-based diagnostics for brain tumors [20]. This paper reviews different types of brain tumors, introduces imaging modalities such as CT, MRI, and PET, and provides an overview of classification and segmentation methods. It includes a literature review and discussion and highlights some challenges.

Ahamed et al. [22] conducted a review of deep learning methods for brain tumor segmentation, offering several valuable observations. Notably, most researchers predominantly utilize the BraTS dataset, thus dealing with only large primary tumors with a large number of training samples. Another conclusion is that the application of fusion and attention mechanisms has been shown to enhance segmentation performance. Therefore, it is expected that a model with attention would be preferable.

The review by Ahamed et al. [22] highlights that detecting small tumors, particularly metastatic ones, has not received much attention. Additionally, the review indicates that recently published papers typically use datasets with at least several hundred cases. Unfortunately, medium-scale hospitals often cannot afford to collect and annotate a large number of brain MRI cases, as carried out in the BraTS challenge. Therefore, it is important to explore how a small dataset can be used to segment and detect small metastatic tumors and to examine the results.

## 3. Methodology

This section describes the experimental datasets, preprocessing steps, experimental models, and conducted experiments. Section 3.1 covers the MRI datasets used. Since the obtained dataset cannot be directly used as training samples, preprocessing steps are detailed in Section 3.2. Section 3.3 discusses the architectures of the chosen models, namely U-net and Swin UNETR. Next, Section 3.4 outlines the parameters used for data augmentation. Section 3.5 describes the three conducted experiments in detail.

### 3.1. MRI Datasets

#### 3.1.1. BraTS2021 Dataset

The BraTS challenge dataset has evolved since its inception in 2012. This paper utilizes the latest version, the BraTS 2021 dataset [26,27,28,29], for Task 1 (Segmentation). As noted by Ahamed et al. [22], BraTS is the dataset most commonly used by researchers. Although other datasets are available, they have various limitations. For example, the Kaggle dataset [25] includes only 2D images. The TCGA-GBM dataset [34] contains 3D images but has only 262 cases. Additionally, the collection of openly available datasets by the University of Cambridge [24] is not specifically designed for brain tumor segmentation.

The BraTS 2021 dataset includes 1251 multimodal MRI cases of primary brain tumors. The BraTS challenge is jointly organized by the Radiological Society of North America (RSNA), the American Society of Neuroradiology (ASNR), and the Medical Image Computing and Computer Assisted Interventions (MICCAI) Society. The data come from multiple medical institutions in countries such as the United States, Germany, Switzerland, Canada, Hungary, and India. All tumor data were manually annotated by one to four experts following the same protocol and finally certified by an experienced committee and approved by neuroradiologists. The dataset is publicly available in [26].

Each case in the BraTS 2021 dataset includes four modalities: T1, T1Gd, T2-weighted, and T2 fluid-attenuated inversion recovery (T2-FLAIR). Each modality has a data size of 240 × 240 × 155 and shares tumor segmentation labels. The segmentation labels are (0, 1, 2, 4), where label 0 represents the background, label 1 represents the necrotic tumor core (NT), label 2 represents the peritumoral edema (ED), and label 4 represents the enhancing tumor (ET). We consider labels 1, 2, and 4 as the entire tumor area. All four modalities of the cases have undergone spatial registration, interpolation to the same resolution, and skull stripping. The final data are stored in the NifTI (.nii.gz) format. Due to usage restrictions stated on the website [26], we officially declare that this dataset is only used for the publication of this paper and not for any other purposes.

#### 3.1.2. The CHGH Dataset

Another set of brain MRI data used in this paper is provided by Cheng Hsin General Hospital (referred to as the CHGH dataset). It was approved by the Institutional Review Board of Cheng Hsin General Hospital, Taipei, Taiwan with the protocol code: (1123)113-53, approval date: 9 October 2024. The tumor areas of the patients were annotated/validated by a neurologist (Y.H.L, one of the authors). The provided MRI data are in the original DICOM (Digital Imaging and Communications in Medicine) [35] format without any processing. The MRI data include images from different planes: axial, coronal, and sagittal, and contains multiple modalities. In this paper, we will use the axial T2-FLAIR modality for experiments, and the tumor area information is stored in the JSON (JavaScript Object Notation) format. The MRI DICOM files and JSON files require additional processing steps to be used in the experiments, which will be detailed in the next subsection.

Table 1 shows the CHGH dataset, which includes 18 large-tumor cases, 15 small-tumor cases, and 22 normal cases. The main task of this paper is to segment and detect small tumors. In the literature, large brain metastases are typically defined as lesions greater than 2 cm in diameter [36]. However, some cases in our study present very long and thin sections of tumors, making it difficult to use diameter as a true representation of tumor size. Therefore, we use the tumor area in a slice, rather than diameter, to classify lesions as “large” or “small.” In this study, a small tumor is defined as a tumor with an area of less than 3.5 cm^2^ in the slice with the largest tumor area in all slices of a case. This threshold is slightly greater than the area of a circle with a diameter of 2 cm. The median area of the small tumors is approximately 1.1 cm^2^, while the median area of the large tumors exceeds 16 cm^2^. The dataset containing only small-tumor cases is denoted as CHGH_S.

### 3.2. Preprocessing of MRI Dataset

#### 3.2.1. Preprocessing of the BraTS2021 Dataset

The dimensions of the input images for the experimental model must be multiples of 32. This means both the size of the images and the number of images in a case should be divisible by 32. The tensor size of the BraTS2021 dataset is 240 × 240 × 155, so we need to adjust it to 128 × 128 × 64. To decimate the slices, we use the following equation:

(1) $$u_{i}=s_{16+2i}$$

where u represents one of the target 64 slices and s represents one of the 155 slices in the BraTS2021 source slices. Since the first 15 slices in the BraTS2021 dataset are blank, we start from the 16th slice and take every other slice until we have 64 slices.

The original BraTS2021 dataset has four labels: 0, 1, 2, and 4. Label 0 is the background, and the other labels represent different tumors. In our experiment, we do not perform detailed tumor segmentation, so we merge labels 1, 2, and 4 into label 1.

#### 3.2.2. Preprocessing of the CHGH Dataset

**Figure 2:**
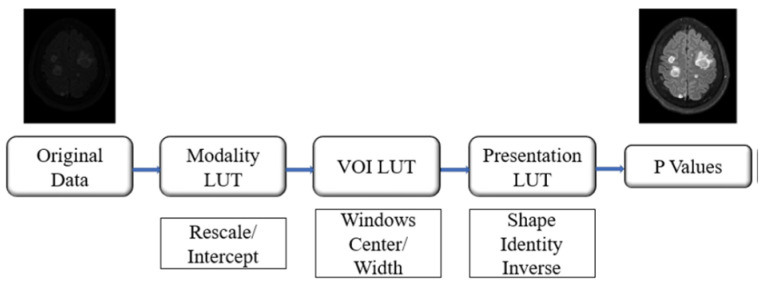
Image preprocessing of the DICOM conversion.

The CHGH MRI data are originally in DICOM format with multiple modalities. For the following experiments, we select the T2-FLAIR as the experimental data. By stacking the processed FLAIR images in sequence, we create a three-dimensional data structure and save it as a NifTi (.nii.gz) file. The tumor location information, marked by the doctor, is originally stored in a JSON file. We need to convert this location information into images and save them as NifTi files as well. Therefore, the final output of each case is a pair of NifTi files, one for the brain image and one for the tumor label. The detailed processing steps are as follows:

- Parsing DICOM files: A DICOM file stores the pixel information of the image and related metadata, such as patient ID, study, series, equipment, and image information. We need to parse the series information part, which contains information related to the series. We use the Pydicom package to parse the DICOM files and the dcmread() function provided by Pydicom to read the DICOM files and access the series description attribute. This makes it very convenient to classify the series. A detailed description of all DICOM attributes can be found on the DICOM official website [35].
- Image pixel conversion: The grayscale range in the original DICOM file images is not uniform, as equipment from different manufacturers usually has minor differences in image details. To address this issue, we need additional steps, as shown in Figure 2.
  1. Modality look-up table (LUT) conversion: This step converts the original grayscale values of the image into a standard space to ensure that images generated by different equipment have consistent measurement standards. We use the DICOM attributes rescale slope (*m*) and rescale intercept ($b_{0}$) to calculate the standardized pixel value (v) from the original pixel value ($v_{org}$) as follows:
    (2) $$v={m\cdot v}_{org}+b_{0}.$$
  2. Value of interest (VOI) LUT conversion: This step primarily aims to enhance image contrast by scaling grayscale values to a windowed range. The VOI is an attribute in the DICOM standard [37], and this conversion is a widely used preprocessing step [38,39]. It uses the DICOM attributes window center/level and window width to perform the grayscale conversion as follows:
    (3) $$G(v)=\left\{\begin{array}{ll} 0, & v<(c-\frac{w}{2})\\ \frac{g_{m}}{w}\left(v+\frac{w}{2}-c\right), & -\frac{w}{2}\leq v\leq (c+\frac{w}{2})\\ g_{m}, & v>(c+\frac{w}{2}) \end{array}\right.$$
    where v is from (2), G(*v*) represents the value after conversion, $g_{m}$ is the maximum display value of the monitor, w is the window width, and c is the window level. Although we did not use a monitor to observe the images, performing this step effectively adjusts the image contrast. In a sense, it serves as another type of grayscale normalization.
  3. Presentation LUT conversion: In this step, we normalize the images to 12-bit grayscale images.
- Parsing JSON annotation files and converting them to image files: In the JSON file we received, the tumor areas are represented by closed lines, composed of continuous segments. Each segment consists of a set of coordinates and width. Using the coordinate information, we draw lines on a blank image canvas, fill the inside of the closed lines with a gray value, and finally save the image. The pairing between the brain image files and the annotation files is matched using the DICOM SopInstanceUID attribute and the JSON file name.
- Interpolation of brain images and tumor annotation images: Although most cases have between 48 and 51 brain slices, a few cases have only 24 or 25 slices. Therefore, we interpolate these brain slices and the paired annotation images to double the slice number, from 24–25 slices to 47–48 slices. This way, the number of slices for all cases will range from 47 to 51 slices. We use the high-order slice interpolation method for interpolation [40,41] because this method outperforms linear and cubic interpolation counterparts.
- Padding black images: Since the number of slices in all cases ranges from 47 to 51, we pad the end of the slices with black images to reach a total of 64 slices. Finally, we save the three-dimensional data structure as a NifTi file with dimensions 128 × 128 × 64.

### 3.3. Experimental Models

In this section, we introduce the experimental models. During the initial research stage, we explored various models, including convolutional neural networks, conventional autoencoders, and ResNet-based networks. However, these models did not yield satisfactory results. We ultimately found that U-net [16,42] and Swin UNETR [17,33] performed better. Therefore, we will focus on describing these two models to save space. For a comprehensive review of U-net and its variants in medical image segmentation, please refer to [43].

#### 3.3.1. U-Net Model

**Figure 3:**
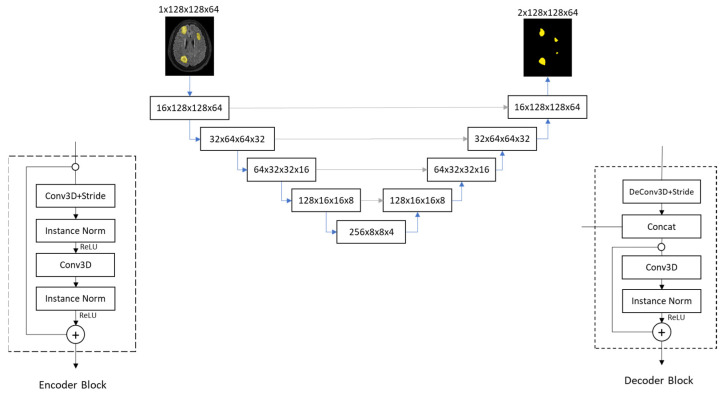
Block diagram of the U-net used in the experiments.

In the following experiments, our U-net model follows the work of Ronneberger et al. [16], as shown in Figure 3. The model input is an image with a size of 1 × 128 × 128 × 64 (Channel × Height × Width × Depth). It first passes through a convolutional layer with a stride of 1, increasing the number of channels to 16, resulting in a feature map size of 16 × 128 × 128 × 64.

Next, the feature map goes through the encoder blocks. Inside each encoder block, as shown at the bottom left of Figure 3, the input first passes through a 3D convolutional layer with a stride of 2, followed by instance normalization and a ReLU (Rectified Linear Unit) activation function. Then, it passes through another 3D convolutional layer with a stride of 1, followed by instance normalization and a ReLU activation function before outputting. It is worth noting that there is a “bypass path,” similar to the design of a residual network [44], connecting the input and output of the encoder block. In the encoding path, the number of channels doubles, and the height, width, and depth are halved from top to bottom blocks. After the 5th encoder block, we obtain a feature map size of 256 × 8 × 8 × 4, which is then fed into the 1st decoder block.

Inside each decoder block, as shown at the bottom right of Figure 3, the input first passes through a 3D transposed convolutional layer with a stride of 2. The output of the transposed convolution is concatenated with the corresponding encoder output (as shown by the gray arrows connecting left and right in Figure 3). Then, it passes through a 3D convolutional layer with a stride of 1, followed by instance normalization and a ReLU activation function. The output of the concatenated layer in a decoder block is connected to the final layer output inside a block, again similar to a residual network [44]. With decoder blocks going upwards, the number of channels is halved, and the height, width, and depth are doubled. After the 4th decoder block, we obtain an output size of 16 × 128 × 128 × 64.

Finally, it passes through a final 3D convolutional layer with a stride of 1, reducing the number of channels to 2, resulting in a tensor size of 2 × 128 × 128 × 64. We then perform an argmax operation on the result, taking the index of the maximum value, and ultimately obtain a tumor segmentation image size of 1 × 128 × 128 × 64.

#### 3.3.2. Swin UNETER Model

**Figure 4:**
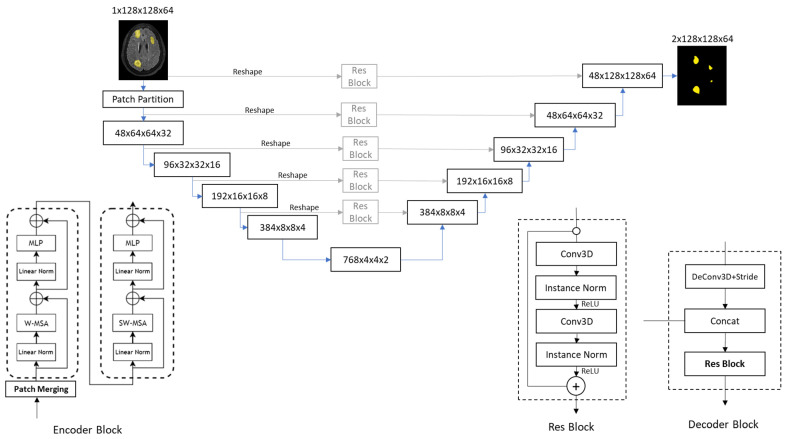
Block diagram of the Swin UNETER model used in the experiments. Note that the linear norm is actually layer norm. However, we follow the drawing of [17] without modification.

In the experiments, the Swin UNETR model consists of five encoder blocks and five decoder blocks, as shown in Figure 4, following the design of [33]. The structure of these five encoder blocks is the same as the Swin Transformer architecture (shown at the bottom left of Figure 4) [31]. The input to the Swin UNETR model is an image with a size of 1 × 128 × 128 × 64 (Channel × Height × Width × Depth). Following the concept of the Swin Transformer, the input image is divided into multiple non-overlapping 3D patches through patch partition and then fed into a series of encoder blocks (shown at the bottom left of Figure 4).

The first step of each encoder block is patch merging, which doubles the number of channels and halves the image resolution, similar to the setup of a convolutional neural network. Each encoder block implements a Swin Transformer, which contains two attention sub-blocks. The first sub-block has a layer normalization, windowed multi-head self-attention (W-MSA), a second layer normalization, and a multi-layer perceptron (MLP, essentially a fully connected multi-layer network). The second sub-block is similar to the first sub-block but uses SW-MSA (shifted windowed multi-head self-attention), instead of W-MSA. After passing through five encoder blocks, we obtain a feature map size of 768 × 4 × 4 × 2.

As shown in Figure 4, there are five decoder blocks in the decoding path. The input to each decoder block first passes through a 3D transposed convolutional layer with a stride of 2, then concatenates with the feature map output from the encoder and finally passes through a residual sub-block before outputting. The feature map from the encoder is reshaped to the same dimension as the decoder block, and then concatenated with the decoder block after passing through the residual sub-block. After the 5th decoder block, we obtain an output size of 48 × 128 × 128 × 64.

Finally, it passes through a final 3-D convolutional layer with a stride of 1, reducing the number of channels to 2. By using the same treatments as in the U-net, we have a tumor segmentation image size of 1 × 128 × 128 × 64.

### 3.4. Data Augmentation

We perform data augmentation in some experiments to expand the number of training samples. By applying various transformations to the original training data, additional samples are generated. Overall, we have used the following transformations:

- Rotation: Rotate the image by 20°~50° or −20°~−50°.
- Scaling: Scale the image by 0.6~0.9 times or 1.1~1.5 times.
- Rotation and scaling: Apply both rotation and scaling with the same range as above.
- Affine transformation: Apply a shear transformation of 0.4 to the image.

Once the augmented samples are generated, they are added to the training set to train the models. For self-supervised learning, the samples for pretext tasks and downstream tasks differ. In this case, the augmentation is performed on training for the downstream models. However, the test set is not subjected to the augmentation step.

### 3.5. Conducted Experiments

Three experiments are conducted in this paper. The first experiment compares the segmentation performance of large and small tumors, measured by the Dice score. The second experiment evaluates the Dice scores of various approaches used to improve the segmentation performance of small tumors. The third experiment computes the sensitivity and specificity of the best model obtained in the second experiment.

#### 3.5.1. Experiment One

We will use the models introduced in Section 3.3.1 and Section 3.3.2 to perform 3-fold cross-validation on the datasets. The training and test datasets are the BraTS2021 and CHGH datasets (only using the 33 tumor cases). The training and testing dataset combinations are as follows:

- Training: BraTS2021; Testing: BraTS2021.
- Training: BraTS2021; Testing: CHGH.
- Training: CHGH; Testing: CHGH.
- Training: BraTS2021 + CHGH; Testing: CHGH.

For using the BraTS2021 dataset for training and testing, the conventional 3-fold cross-validation is carried out. For using the CHGH as the test set, we first divide the CHGH dataset into three subsets: O1, O2, and O3, each containing 11 cases. Next, we select the O1 subset as the test set and the remaining subsets, if applicable, as part of the training data. We then conduct complete supervised learning and evaluate O1. This process is repeated for O2 and O3.

#### 3.5.2. Experiment Two

**Figure 5:**
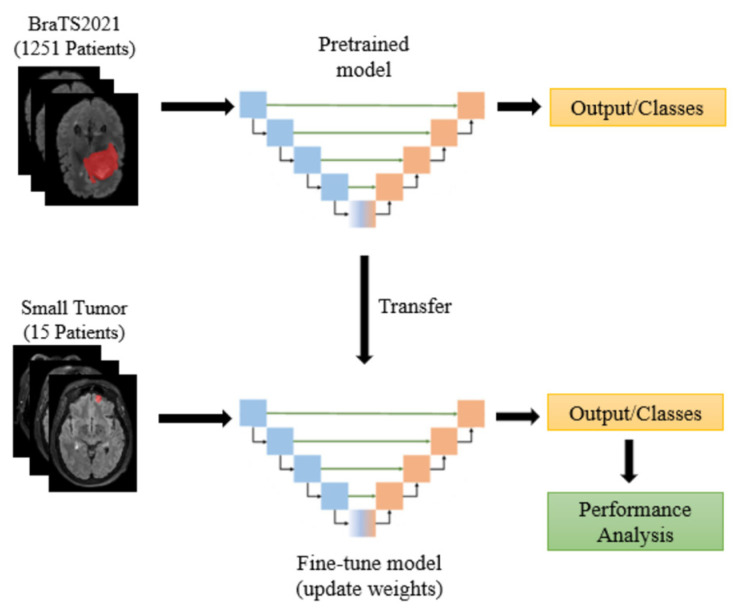
Fine-tuning of the pretrained model in the experiments. The encoder part consists of blue blocks and the decoder part consists of the orange blocks.

**Figure 6:**
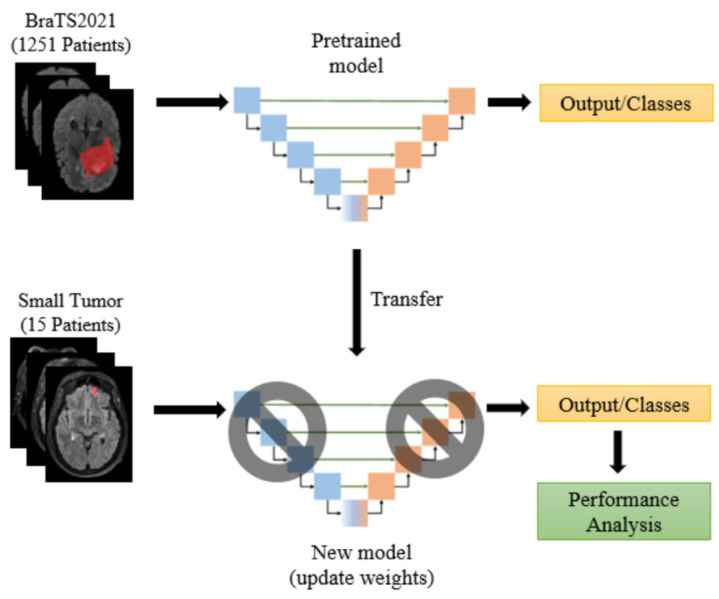
Layer-freezing approach of transfer learning in the experiments. The encoder part consists of blue blocks and the decoder part consists of the orange blocks.

**Figure 7:**
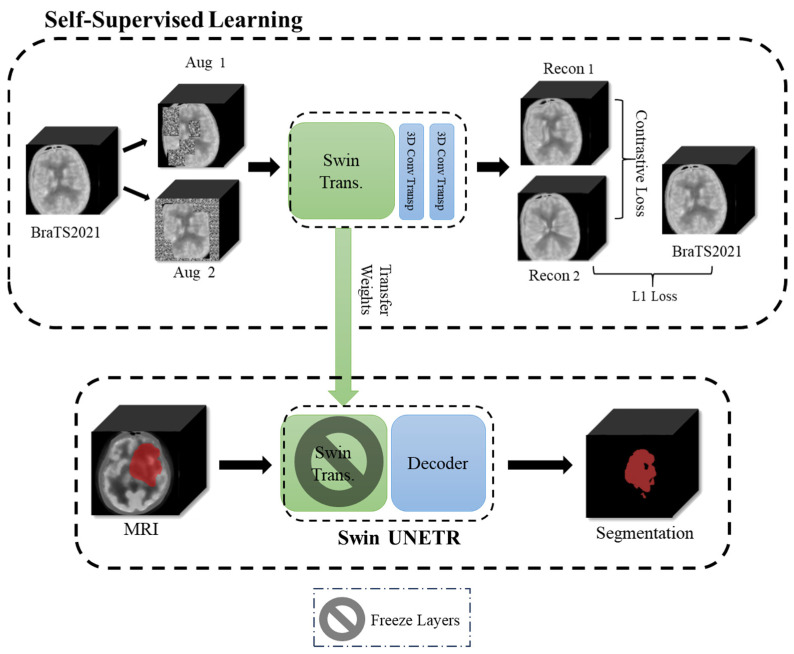
Self-supervised learning approach used in the experiments.

The BraTS2021 training set consists of 1251 patients, primarily with large and prominent tumors. In contrast, the CHGH dataset includes 33 cases, of which 18 have large tumors and the remaining 15 have small tumors. With such a small number of small-tumor cases, it is difficult to train a model to achieve higher performance. We will show that Swin UNETR has a higher performance in Experiment One. Therefore, this experiment only uses Swin UNETR. As the number of small-tumor cases is limited, we perform leave-one-out cross-validation to report the segmentation results. To find an effective segmentation method for small tumors, we study the following five methods:

- Supervised learning: This method uses the BraTS2021 + CHGH_S data as the training set with conventional supervised learning. Recall that leave-one-out cross-validation is used for CHGH_S during testing. Therefore, all cases but one in CHGH_S are in the training set. This method does not use an augmented dataset during training due to the large number of training samples and the limitations of the experimental hardware on training the augmented dataset.
- Supervised learning with data augmentation: The training set is CHGH_S with different augmentation rates to observe whether data augmentation is useful for this problem.
- Transfer learning with parameter finetuning: Pre-train the Swin UNETR model using the BraTS2021 dataset with supervised learning, then use transfer learning to fine-tune the parameters with augmented CHGH_S cases, as shown in Figure 5. Specifically, we first use the BraTS2021 training set to conduct complete supervised learning to obtain a pretrained model. We then use the weights of the pretrained model to fine-tune the model with augmented CHGH_S training data. For finetuning, it is important to select the appropriate number of training epochs. Therefore, we use 2, 4, 6, 8, and 10 epochs to fine-tune the model with four-times augmentation rates and select the number of epochs that perform best. With this epoch number, the performance of other augmentation rates is examined.
- Transfer learning with layer freezing: This method also pre-trains a model using the BraTS2021 dataset. However, during fine-tuning, we only train the bottom two layers, freezing the weights of all other layers, as shown in Figure 6. We hope that the top layers of the model can learn to extract features from the BraTS2021 dataset while allowing the bottom two layers to adapt to segment small tumors. This approach aims to enhance the model’s generalization performance.
- Self-supervised learning: Pre-train the model using the BraTS2021 dataset with self-supervised learning (SSL), then use different augmentation rates on small-tumor cases for downstream training. The training procedure is shown in Figure 7. Following the procedure of [32,33], we use the encoder (i.e., Swin Transformer) part of Swin UNETR to perform contrastive learning on the BraTS2021 dataset. We apply an inner cutout and outer cutout to generate contrastive images as the inputs to the pretext task.

The inner cutout images are obtained by randomly cropping the interior of the image with sizes ranging from a minimum of 5 × 5 to a maximum of 32 × 32 and adding noise. The number of crops is 6.

The outer cutout images are obtained by randomly cropping the outer edges of the image with sizes ranging from a minimum of 20 × 20 to a maximum of 64 × 64 and adding noise. The number of crops is also 6.

The resulting images, referred to as Aug 1 and Aug 2 in Figure 7, are the inputs to the Swin Transformer model, producing reconstructed images Recon 1 and Recon 2. We calculate the contrastive loss between Recon 1 and Recon 2, as well as the L1 Loss between Recon 2 and the original, un-augmented data. The combined loss from the contrastive loss and L1 Loss is used to update the model’s weights. For the downstream task, only augmented data from small-tumor samples are used in training. During the downstream training, the encoder parameters are fixed and only the decoder parameters are trainable.

#### 3.5.3. Experiment Three

We use the best-performing model from Experiment Two to predict cases with and without small tumors, evaluating the sensitivity and specificity of the trained model.

## 4. Experimental Results

### 4.1. Experimental Environments

Experiments are conducted through computer simulations using two computers. The hardware specifications and software package versions are provided in Table 2 and Table 3, respectively. Although these two computers have different hardware and software configurations, we conducted trial experiments to ensure they produced exactly the same results. Using two computers allows the experiments to be completed faster.

### 4.2. Results for Experiment One

**Figure 8:**
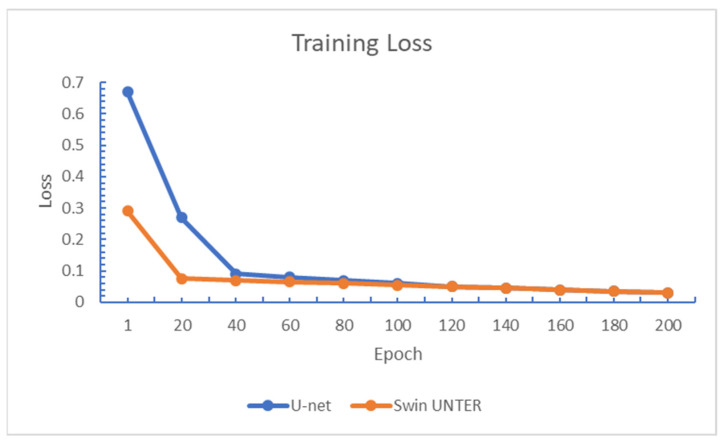
Training loss of both models in Experiment One.

**Figure 9:**
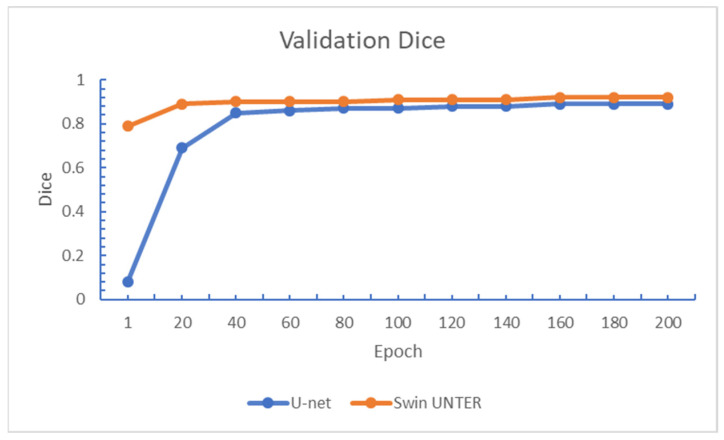
Validation Dice scores of both models in Experiment One.

This experiment compares the relative performance of the U-net and Swin UNETR, in terms of convergence speed and testing Dice scores. Figure 8; Figure 9 show the changes in training loss and validation Dice scores over training epochs for the BraTS2021 + CHGH training set. It is observed that Swin UNETR converged faster than U-net. However, after about 50 epochs, the convergence speed of both models became roughly the same. In terms of validation Dice, the scores of both models increased more slowly after 20 epochs compared to before. After 100 epochs of training, the loss value for both models dropped to around 0.03. In terms of validation Dice scores, U-net can reach a maximum score of around 0.88, while Swin UNETR can reach around 0.91.

The results of the three-fold cross-validation are shown in Table 4. It is observed that the Dice scores of Swin UNETR on the BraTS2021 test set and the CHGH test set are about 3% higher than those of U-net using the BraTS2021 dataset as the training set. For the training set including a portion of the CHGH data, Swin UNETR is about 6.3% higher than U-net. On average, the Dice scores of the Swin UNETR model are 4–5% higher than its counterpart.

It is also observed that the Dice scores for segmenting the CHGH test set are lower than those for the BraTS2021 test set. This is because, among the 33 cases in the CHGH dataset, 15 have only small tumors. The average Dice score for segmenting large tumors is 0.78, whereas the average Dice score for segmenting small tumors is almost zero (0.03 in Table 4), even with Swin UNETR. This indicates that the models used cannot segment small tumors satisfactorily, thus affecting the average Dice score of the CHGH dataset. As the BraTS2021 dataset does not contain any samples with small tumors, the models trained with this dataset perform poorly when segmenting small tumors. To improve performance, we study several methods mentioned in Experiment Two (Section 3.5.2).

### 4.3. Results for Experiment Two

This experiment compares various approaches to train Swin UNETR, including conventional supervised learning (SL), finetuning and layer freezing of transfer learning, and self-supervised learning (SSL). In the case of finetuning, it is found that the accuracy is highest with 6 epochs of training. Therefore, only the results of 6 epochs are reported to save space. The experimental results are given in Table 5.

It is observed that, as mentioned previously, adding the BraTS2021 dataset to the training set provides very little improvement (0.0169 vs. 0.0174) compared to training only with the CHGH_S dataset. It is also evident that using data augmentation can significantly improve the Dice scores. However, using data augmentation in supervised learning can only achieve a Dice score of 0.13, which is comparable to that of transfer learning.

From Table 5, we can see that the self-supervised learning model has a higher Dice score than other approaches before applying data augmentation. With augmentation rates of 4, 8, or 16 times, the Dice scores of self-supervised learning also show a clear advantage. Additionally, when the augmentation rate increases from 8 times to 16 times, the performance of supervised learning and transfer learning shows no improvement, whereas the performance of self-supervised learning continues to show an upward trend until it reaches its limit at 16 times. In fact, further increasing the augmentation rate to 32 times reduces the Dice score. Therefore, the self-supervised learning model with an augmentation rate of 16 times is the one to be used in Experiment Three.

**Figure 10:**
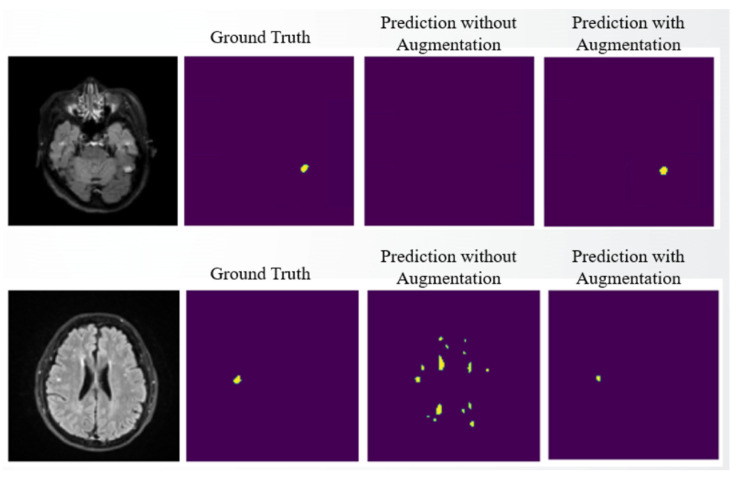
Performance improvement with data augmentation. The yellow regions are the tumor regions marked by different methods.

The increase in the Dice score with data augmentation actually helps tumor detection, in both sensitivity and specificity. Taking the subject at the top of Figure 10 as an example, without using data augmentation, the small tumor was not detected, but after applying data augmentation, the small tumor was correctly detected. Thus, sensitivity improves. Conversely, for the subject at the bottom of Figure 10, there were segmentation errors without using data augmentation. But after applying data augmentation, the falsely detected tumors disappear, thereby improving specificity. In short, applying data augmentation is an essential step in training the above models.

### 4.4. Results for Experiment Three

This experiment studies the sensitivity and specificity of Swin UNETR trained with self-supervised learning and performing data augmentation to generate 16 times the amount of data. In this experiment, we performed tumor detection on all subjects in the CHGH dataset, including the 22 normal cases. Please note that normal cases were not used during training. The results, given in Table 6, show that the model has 100% accuracy in detecting small tumors. However, in terms of specificity, 10 subjects were incorrectly detected with tumors. Therefore, the specificity of this model is 54.5%.

**Figure 11:**
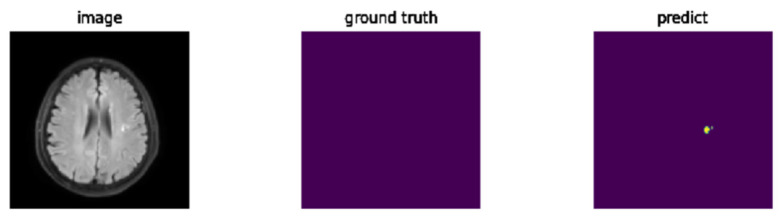
A normal subject with hyperintensities. The yellow region on the right picture is due to a misclassification of the model.

Among the 10 incorrectly detected cases, 7 of them have white bright spots in their MRIs. After reviewing the subjects’ medical records, these white bright spots were confirmed as white matter hyperintensities rather than actual tumors, as shown in Figure 11. However, it is difficult, even for an expert, to distinguish whether these white spots are tumors or not using only MRI. Therefore, to improve the model’s specificity, additional information beyond MRI, such as the subject’s medical records, is needed in an actual diagnosis. Nevertheless, if we exclude subjects with white spots, the model’s specificity is 12/15 = 80.0%.

### 4.5. Comparison with Existing Works

In terms of segmentation results, we could not find any existing literature discussing the segmentation of small tumors. Although we found papers addressing segmentation performance on BraTS 2021, such as [45,46], our main focus is not on segmenting BraTS 2021 images. So, we will not provide comparison results here. When comparing our findings with the existing research on brain tumor detection by Abdusalomov et al. [19], the results are displayed in Table 7.

It is apparent that the specificity of our results is relatively low compared to those of Abdusalomov et al. [19]. However, several factors are worth noting. Firstly, the experimental dataset used by Abdusalomov et al. is significantly larger than ours (8000+ vs. 32 cases), which certainly contributes to improved detection performance. Secondly, the study by Abdusalomov et al. is not specifically designed for detecting small tumors. As stated in the article’s summary, “we acknowledge that additional investigation and testing are essential to ensure the effectiveness of our method for detecting small tumors”. This acknowledgment underscores the technical challenges of detecting small tumors, particularly given the limited size of our dataset.

## 5. Discussions and Future Directions

Given the proposed approach’s sensitivity of 100%, it ensures that all tumors are correctly detected. High sensitivity is typical, as the model was trained to perform segmentation, which requires accurately finding the locations of tumors. However, the low specificity of 54.5% may not be sufficient for many applications. In a typical situation, using a threshold determined by, say, Youden’s index can trade sensitivity for specificity. To determine the threshold, a validation set is necessary, partitioned from the test set. This process ensures no data (or knowledge) leakage during experiments. That is, no information from the test set is used during the testing phase. Unfortunately, in our case, it is difficult to further partition the validation set from the already small dataset.

The low specificity of 54.5% of our approach is partly due to some cases having white spots on the MRI slices. By excluding these subjects, the specificity increases to 80%, which could be sufficient for screening purposes. Currently, we are investigating using PET-CT (positron emission tomography–computed tomography) images in conjunction with the MRI for the same subject to improve the specificity. Still, the presented experiments confirm the feasibility of detecting small tumors with the proposed method with a small dataset.

In this paper, our dataset is relatively small. While CHGH has many more cases available for study, expanding the experimental dataset is not extremely difficult. However, our intention is to emulate the limited resources of a medium-scale hospital. Therefore, we decided to use a small dataset for training and testing, with cases randomly selected to include both large and small tumors. The advantage of training a model with an in-house dataset, despite its small size, cannot be matched by using an openly available dataset, such as BraTS 2021. Using such a dataset may lead to the over-expectation of performance in real-world cases. For example, a model trained with the BraTS dataset reaches a Dice score of 0.90 for segmenting its own samples (in Experiment One), closely matching the latest works that also use BraTS, such as [17,45,46]. Thus, the model is successfully trained. However, when this model is used to segment small tumors, the score drops to 0.0332, indicating that it cannot hope to have any useful medical applications.

In our experiments, the model using the proposed self-supervised learning approach improved the Dice score to 0.1882. While this score is still low, the trained model is nevertheless useful, as demonstrated in the detection experiment with acceptable results. Therefore, the model could be used for the initial screening of MRI scans to detect tumors and pinpoint their locations, reducing the workload of neurologists.

The feasibility of using a small in-house MRI dataset for brain tumor detection and segmentation also opens up the possibility of exploring other types of diseases, such as stroke. There are open MRI datasets for stroke lesion segmentation, such as [47]. By using a similar method presented in this paper, a hospital may develop a model tailored to a specific application on stroke with a small in-house dataset.

It is also important to note that the Dice score tends to be lower for small objects. The Dice score is calculated as follows:

(4) $$Dice=\frac{2T_{P}}{2T_{P}+F_{P}+F_{N}}$$

where $T_{P}$ is the region of true positives, $F_{P}$ is the region of false positives, and $F_{N}$ is the region of false negatives.

**Figure 12:**
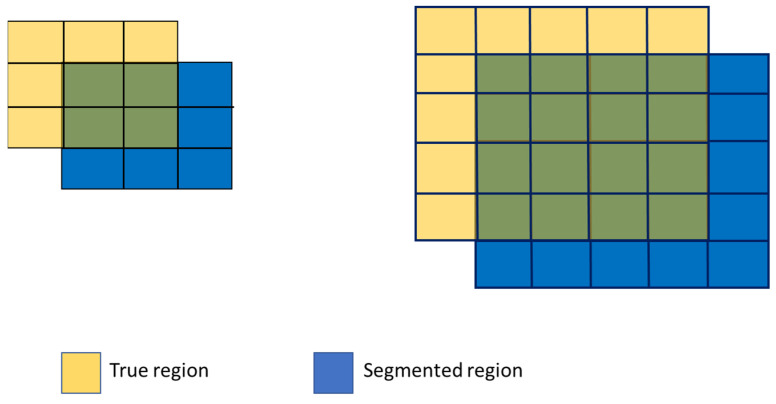
Example of influences of segmentation offset on Dice score for different sized ROIs. The overlapped parts are shown in green.

To illustrate that the Dice score favors larger regions over smaller ones, consider the following hypothetical example in Figure 12: a task to segment a small region size of 3 × 3 and a large region size of 5 × 5. Due to technical reasons (such as labeling bias, misalignment, etc.), the segmented regions are offset by one pixel in both horizontal and vertical directions. According to (4), the Dice score for the small region is as follows:

$$\frac{2\times 4}{2\times 4+5+5}=0.44.$$

On the other hand, the Dice score for the large region is as follows:

$$\frac{2\times 16}{2\times 16+9+9}=0.64.$$

If the region is larger, the score is even higher. In practical cases, the labeled ground truth rarely coincides exactly with the tumor. Offsetting by one or two pixels in each direction is common. In our case, some small tumors have areas less than 1 ${cm}^{2}$ in one slice. As we resample the images to a size of 128 × 128, an offset of a couple of pixels in segmentation (or annotation) significantly affects the Dice score, making a low Dice score reasonable.

In the future, we plan to implement a two-step approach to improve the Dice score. The first step involves cutting a portion of an MRI slice containing the region of interest (ROI). The second step is to enlarge the ROI portion to increase the number of pixels representing the small tumors. A similar approach for lung cancer is used in [48]. The segmented ground truth should be based on the enlarged ROI. This method should be able to increase the Dice score.

As mentioned in the introduction, detecting small tumors and continuously monitoring their growth rate is beneficial. Therefore, another future direction would be to develop an algorithm to automatically compute the growth rate of tumors based on several sets of MRIs taken on different dates.

## 6. Conclusions

This paper studies the segmentation and detection of small metastatic brain tumors using a small set of MRI scans. The available literature primarily focuses on segmenting or detecting large primary brain tumors with a large number of training cases. Our study closely resembles a real scenario that a medium-scale hospital may face: a wide variety of tumor sizes and types (primary or metastatic), but insufficient resources to create a large in-house dataset.

When comparing the performance of the U-Net and Swin UNETR models, the latter performs better in tumor segmentation tasks. Even so, our experimental results show that using the BraTS 2021 dataset to train a Swin UNETR model still yields poor performance in segmenting small tumors. To address this, we use self-supervised learning, which has two training phases: pretext task training and downstream task training. By using the BraTS dataset for pretext task training, we can leverage the large number of samples in the dataset to aid the downstream task. Overall, self-supervised learning with data augmentation offers advantages in terms of Dice scores, from 0.0332 to 0.1882.

When directly using the self-supervised model, designed for segmentation, to perform small tumor detection, experimental results show good sensitivity (100%) and acceptable specificity values (80% if excluding cases with white matter hyperintensities). Overall, the experiments confirm the feasibility of segmenting and detecting small tumors with only a small number of in-house samples. To further improve the model’s specificity for subjects with white matter hyperintensities, additional information beyond MRI scans is needed in the detection model.

## Figures and Tables

**Table 1:** The CHGH datasets.

Dataset	CHGH
Large Tumor Subject	18
Small Tumor Subject	15
No Tumor Subject	22 ^1^

^1^ Used only in Experiment three.

**Table 2:** Specifications of the computers used in the experiments.

Item	Computer 1	Computer 2
Processor	Intel Core i9-9900x@3.50 GHz	Intel Core i7-6700@3.40 GHz
Memory	32 GB DDR4	32 GB DDR4
Display Card	NVIDIA GTX 1080-Ti	NVIDIA GTX 1080-Ti
Mother Board	Gigabyte X299 Designer EX	ASUS 820MT
OS	Ubuntu 20.04.6 LTS	Windows 10 Pro

**Table 3:** Versions of software packages used in the experiments.

Package	Version
Python	3.10
Pytorch	2.0.0
CUDA	11.6
MONAI	1.2.0

**Table 4:** Dice scores in Experiment One.

Training/Test	U-Net	Swin UNETR
BraTS2021/BraTS2021	0.8725	0.9036
BraTS2021/CHGH	0.2574	0.2871
CHGH/CHGH	0.2863	0.3500
BraTS2021 + CHGH/CHGH	0.3565	0.4402
BraTS2021 + CHGH/CHGH_S	-	**0.0332**

**Table 5:** Dice scores in Experiment Two.

Training/Downstream	Method	Augmentation Rate			
		×1	×4	×8	×16
BraTS2021 + CHGH_S	SL	0.0174	-	-	-
CHGH_S	SL	0.0169	0.1203	0.1270	0.1258
BraTS2021/CHGH_S	Finetuning	0.0285	0.1109	0.1220	0.1260
BraTS2021/CHGH_S	Layer Freezing	0.0135	0.0662	0.1195	0.1172
BraTS2021/CHGH_S	SSL	**0.0600**	**0.1439**	**0.1517**	**0.1882**

**Table 6:** Detection results.

Subject	Tumor Detected	Not Detected
With small tumors (15 cases)	15	0
Normal with hyperintensities (7 cases)	7	0
Normal without hyperintensities (15 cases)	3	12

**Table 7:** Performance comparison.

Approach	Training	Test	Tumor Size	Sensitivity	Specificity
Abdusalomov et al. [19]	8232	2056	Large	0.99	0.99
Proposed	32 ^1^	15 + 22	Small	1.0 ^2^	0.55/0.8 ^3^

^1^ Model trained for segmentation with BraTS 2021 for training the pretext task. ^2^ Based on leave-one-out cross-validation. ^3^ Excluding cases with white matter hyperintensities.

## Data Availability

The BraTS2021 is publicly available [26]. The CHGH data will be available on February 2025 at https://github.com/NTUT-LabASPL2/BrainMRI.
